# Pharmacological inhibition of HDAC6 reverses cognitive impairment and tau pathology as a result of cisplatin treatment

**DOI:** 10.1186/s40478-018-0604-3

**Published:** 2018-10-01

**Authors:** Jiacheng Ma, XiaoJiao Huo, Matthew B. Jarpe, Annemieke Kavelaars, Cobi J. Heijnen

**Affiliations:** 1 Laboratory of Neuroimmunology, Department of Symptom Research, The University of Texas MD Anderson Cancer Center, 1515 Holcombe Blvd, Unit 384, Houston, TX 77030 USA; 2 Regenacy Pharmaceuticals Inc., Boston, MA 02210 USA

**Keywords:** Chemotherapy-induced cognitive impairment, HDAC6, α-Tubulin, Tau, Mitochondria, Synaptic integrity

## Abstract

Chemotherapy-induced cognitive impairment (CICI) is a commonly reported neurotoxic side effect of chemotherapy, occurring in up to 75% cancer patients. CICI manifests as decrements in working memory, executive functioning, attention, and processing speed, and greatly interferes with patients’ daily performance and quality of life. Currently no treatment for CICI has been approved by the US Food and Drug Administration. We show here that treatment with a brain-penetrating histone deacetylase 6 (HDAC6) inhibitor for two weeks was sufficient to fully reverse cisplatin-induced cognitive impairments in male mice, as demonstrated in the Y-maze test of spontaneous alternation, the novel object/place recognition test, and the puzzle box test. Normalization of cognitive impairment was associated with reversal of cisplatin-induced synaptosomal mitochondrial deficits and restoration of synaptic integrity. Mechanistically, cisplatin induced deacetylation of the microtubule protein α-tubulin and hyperphosphorylation of the microtubule-associated protein tau. These cisplatin-induced changes were reversed by HDAC6 inhibition. Our data suggest that inhibition of HDAC6 restores microtubule stability and reverses tau phosphorylation, leading to normalization of synaptosomal mitochondrial function and synaptic integrity and thereby to reversal of CICI. Remarkably, our results indicate that short-term daily treatment with the HDAC6 inhibitor was sufficient to achieve prolonged reversal of established behavioral, structural and functional deficits induced by cisplatin. Because the beneficial effects of HDAC6 inhibitors as add-ons to cancer treatment have been demonstrated in clinical trials, selective targeting of HDAC6 with brain-penetrating inhibitors appears a promising therapeutic approach for reversing chemotherapy-induced neurotoxicity while enhancing tumor control.

**Electronic supplementary material:**

The online version of this article (10.1186/s40478-018-0604-3) contains supplementary material, which is available to authorized users.

## Introduction

The success of cancer treatment has increased dramatically over the past decade, with the survivor population reaching 15.5 million in the US alone and 28 million worldwide in 2016 [14, 22]. However, the quality of life of cancer survivors is often affected by serious chemotherapy-induced neurotoxic side effects, including chemotherapy-induced cognitive impairment (CICI), also termed “chemobrain” [26, 49]. CICI is reported by up to 75% patients treated for cancer and manifests as decrements in working memory functioning, executive functioning, attention, and processing speed [17, 26]. Prospective neuroimaging fMRI studies have indicated hypoactivation of various brain regions, and diffusion tensor imaging analysis has indicated structural changes in white-matter areas of the brain in patients treated for cancer [16, 28, 36]. To date, no preventive or curative intervention for CICI has been approved by the US Food and Drug Administration. We have therefore developed a model of cisplatin-induced cognitive deficits in the mouse to allow investigation of the mechanism and development of curative interventions for CICI.

A major concern in developing interventions for chemotherapy-induced neurotoxicities is that they might interfere with tumor control. An absolute requirement for the treatment of CICI would be to exclusively use agents that do not interfere with, and preferably even enhance tumor control. In this regard, agents targeting histone deacetylase 6 (HDAC6) are promising candidates to fill this void. HDAC6 is a unique member of the histone deacetylase family, as it does not interact with histones in vivo [21]. Its primary targets include non-histone cytoplasmic substrates, such as the microtubule protein α-tubulin and heat shock protein 90 (HSP90) [25, 30]. Inhibitors of HDAC6 display anti-tumor activity in various cancer models; thus, HDAC6 inhibition is being considered as an add-on treatment to inhibit tumor growth [1, 2, 24, 46]. One of the HDAC6 inhibitors, ACY-1215 (ricolinostat), is currently in multiple clinical trials for evaluation of its anti-tumor activity (NCT01997840, NCT02091063, NCT02632071, NCT01583283, NCT02189343, NCT02787369). Notably, it has already been shown to be safe, well-tolerated, and active in enhancing the efficacy of the combination of bortezomib and dexamethasone for multiple myeloma in a Phase II clinical trial (NCT01323751) [46].

In addition to the anti-tumor activity, pharmacological inhibition or genetic ablation of HDAC6 have been shown to protect against cognitive deficits in rodent models of Alzheimer’s disease (AD) [18, 20, 34]. Mechanistically, HDAC6 inhibition improves neuronal mitochondrial transport [10]. This is interesting because synaptosomal mitochondrial damage is a key underlying mechanism of cisplatin-induced cognitive impairment [11]. Moreover, inhibition of HDAC6 has been shown to normalize cisplatin or vincristine-induced decrease in mitochondrial transport in peripheral sensory neurons [31, 45]. Further, HDAC6 inhibition has been shown to attenuate tau pathology [13, 39, 44], a mechanism closely linked to cognitive decline in neurodegenerative diseases [19, 29, 38]. However, it is not known whether cisplatin-induced cognitive deficits are associated with tau pathology.

In the current study, we tested the hypothesis that inhibition of HDAC6 reverses CICI [31]. We investigated the effects of the brain-penetrating HDAC6 inhibitor ACY-1083 on cisplatin-induced cognitive deficits and neuronal mitochondrial damage. We also investigated the effects of ACY-1083 on synaptic integrity, given our previous data showing reduction of dendritic spines and neuronal arborizations induced by cisplatin that is indicative of loss of synaptic function [54]. Furthermore, we determined the effect of cisplatin and HDAC6 inhibition on tau phosphorylation.

## Materials and methods

### Animals

Male C57BL/6 J mice (aged 8–10 weeks at the start of the experiment) were obtained from Jackson Laboratories (Bar Harbor, ME) and housed in The University of Texas MD Anderson Cancer Center animal facility on a reversed 12-h light/dark cycle, with free access to food and water. Animals were randomly assigned to treatment groups. All procedures were consistent with the National Institute of Health Guidelines for the Care and Use of Laboratory Animals and were approved by the Institutional Animal Care and Use Committee (IACUC) of M.D. Anderson Cancer Center. Analyses were performed by investigators blinded to treatment.

### Drug administration

Mice were treated with cisplatin (2.3 mg/kg/day, TEVA Pharmaceuticals, North Wales, PA) diluted in sterile PBS or PBS alone intraperitoneally (i.p.) for 5 days, followed by 5 days of rest and a second round of 5 daily doses of cisplatin or PBS [31]. The HDAC6 inhibitor ACY-1083 (Acetylon Pharmaceuticals Inc., Boston, MA) was dissolved in 20% 2-hydroxypropyl-B-cyclodextrin (Sigma-Aldrich, St. Louis, MO) + 0.5% hydroxypropyl methylcellulose (Spectrum Chemical, Gardena, CA) in water. Mice received i.p. injections of ACY-1083 at 10 mg/kg. The HDAC inhibitor ACY-1215 (Ricolinostat; Regenacy Pharmaceuticals Inc., Boston, MA) was dissolved in 10% DMSO (Sigma-Aldrich), 30% propylene glycol (Sigma-Aldrich), and 60% PEG-300 (Sigma-Aldrich), and was administered at 30 mg/kg via oral gavage [31].

### In vivo pharmacokinetics

For in vivo pharmacokinetic studies, mice were fasted overnight and i.p. injected with 5 mg/kg ACY-1083 dissolved in 10% dimethylacetamide (DMAC, Sigma-Aldrich, St. Louis, MO) + 10% Solutol HS 15 (BASF, Houston, TX) in saline, or 30 mg/kg ACY-1215 dissolved in 10% DMAC+ 15% Solutol HS 15 in saline. Blood and brain were collected at 5 min, 15 min, 30 min, 1 h, 4 h and 8 h post injection. Plasma was obtained by centrifugation at 2000×*g* for 5 min at 4 °C. Brain homogenates were obtained by homogenizing the brain in 3 volumes of PBS. Plasma and brain compound level was analyzed using liquid chromatography-tandem mass spectrometry (Waters Corporation, Milford, MA) and was calculated from standard curves of ACY-1083 and ACY-1215 in mouse plasma and brain, respectively. Pharmacokinetic parameters were calculated using WinNonlin software (Certara USA, Inc., Princeton, NJ).

### Behavioral testing

**Fig. 1:**
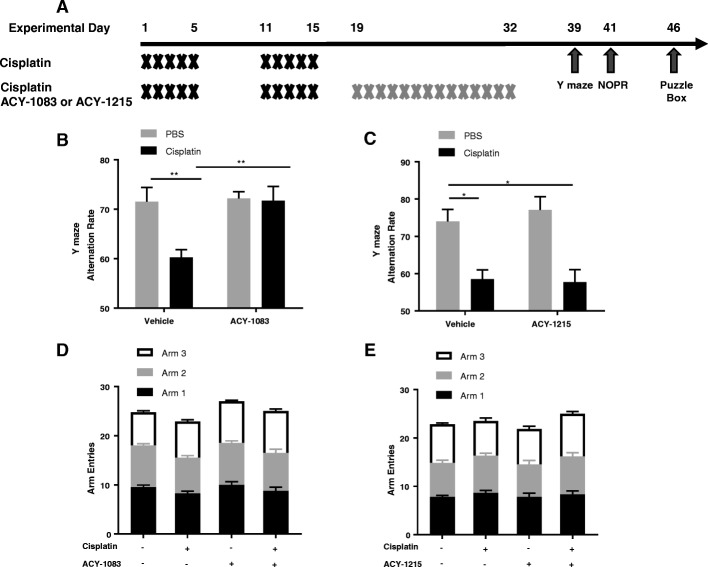
Effect of HDAC6 inhibition on cisplatin-induced cognitive impairment in the Y maze test. **a** Mice were treated with two 5-day cycles of cisplatin or PBS, followed by 14 daily administrations of HDAC6 inhibitor (either ACY-1083 or ACY-1215) or vehicle starting 3 days after the last dose of cisplatin/PBS. Behavioral tests including Y-maze, NOPR, and the puzzle box tests were started one week post the last ACY-1083 injection as indicated in the timeline. The Y-maze test of spontaneous alternations was performed 1 week after the last injection of the two HDAC6 inhibitors. The percentage of perfect alternations (alternation rate) was calculated: (**b**) ACY-1083 (*n* = 8; two-way ANOVA with Tukey’s post-hoc analysis: F (1, 28) = 5.547, *p* = 0.0258; PBS vs. Cisplatin, *p* = 0.0086; Cisplatin vs. Cisplatin + ACY-1083, *p* = 0.0074) or (**c**) ACY-1215 (*n* = 6; two-way ANOVA with Tukey’s post-hoc analysis: F (1, 20) = 0.3809, *p* = 0.5441; PBS vs. Cisplatin, *p* = 0.0148; PBS vs. Cisplatin + ACY-1215, *p* = 0.0096). Total number of arm entries and number of arm entries into each individual arm: (**d**) ACY-1083 (*n* = 8; two-way ANOVA, F (1, 28) = 0.002633, *p* = 0.9594) (**e**) ACY-1215 (n = 6; two-way ANOVA, F (1, 20) = 0.7648, *p* = 0.3922). Results are expressed as means ± SEM; *p < 0.05, **p < 0.01

We used the Y-maze test [23], the novel object/place recognition (NOPR) test [3], and the puzzle box test [5] to assess cognitive function in mice. The tests were conducted starting 1 week after the last dose of ACY-1083 or ACY-1215 treatment. The timeline for the behavioral tests were indicated in Fig. 1a. For the Y-maze test, mice were placed in a symmetrical three-arm, gray plastic Y-maze (35 cm length × 5 cm width × 15.5 cm height per arm, with an arm angle of 120°) with external spatial room cues. Mice were placed in one of the arms, and spontaneous movement was recorded for 5 min. A perfect alternation was defined as exploration of all three arms sequentially without reentering a previously visited arm. All four paws must have been within the arm to be counted as an entrance. Alternation rate, total number of arm entries and the number of entries into each arm were recorded. The alternation rate was defined as the ratio of the number of perfect alternations to the total number of possible perfect alternations [11].

The NOPR test was performed as we have previously described [11]. A testing arena (46.99 cm × 25.4 cm) was set up with two identical objects placed on the same side of the arena. Mice were placed in the testing arena for 5 min (training phase) and then placed back in their home cage. Thirty minutes later, mice were transferred back to the arena, now containing one familiar object placed at the same location as in training, and one novel object placed on the opposite end of the arena (testing phase). Investigation behavioral was defined as nose point within 1 cm of the object. The time (T) of investigative behavior toward either object during the 5-min testing phase was evaluated using EthoVision XT 10.1 video tracking software (Noldus Information Technology Inc., Leesburg, VA). Discrimination index was determined as (T_Novel_ − T_Familiar_)/(T_Novel_ + T_Familiar_).

The puzzle box test was performed 2 weeks after the last injection of ACY-1083, as previously described with slight modification [5]. The testing area consisted of a white box divided by a black barrier into two compartments: a brightly-lit start zone (58 cm × 28 cm) and a smaller, covered goal zone (15 cm × 28 cm) connected by a 4 cm-wide underpass located under the barrier. Mice were introduced into the start zone, and the time it took for the mouse to enter the goal zone was recorded. The test consisted of 11 trials over 4 consecutive days, with two or three trials per day and obstructions of increasing difficulty placed at the underpass on day 2 to day 4. The goal zone was baited with chocolate-scented pellets (ThermoFisher Scientific, Waltham, MA). On day 1 trials 1–3, the underpass was wide open. On day 2, trial 4 was identical to the three trials on the first day. In trials 5 and 6, the underpass was filled with corncob bedding and mice had to dig their way through to get to the goal zone. On day 3, trial 7 was a repetition of trials 5 and 6. In trials 8 and 9, the underpass was obstructed with a cardboard plug that has the same color as the start zone. This task requires a series of events including spatial and short-term memory regarding the position of the underpass, recognition of the cardboard plug that’s obstructing the object, and removal of the plug for which the mice need to employ their teeth and front paws to pull out the obstruction (problem-solving), which is more complex and engage heavier cognitive load. On day 4, trials 10 and 11 were a repetition of trials 8 and 9.

### Western blot analysis

Brains were homogenized in RIPA buffer (50 mM Tris·HCl, pH 7.5, 500 mM NaCl, 2.5 mM MgCl_2_, 1% NP-40, 10% glycerol) containing proteinase inhibitors; 30 μg of protein was separated by SDS/PAGE and transferred to PVDF membrane for immunoblot analyses. The membranes were blocked with 5% nonfat dry milk in PBS containing 0.1% Tween 20 (PBST) and probed with primary antibodies recognizing acetylated lysine (1:2000; rabbit monoclonal; Abcam (ab190479), Cambridge, UK), α-tubulin (1:1000; rabbit polyclonal; Cell Signaling Technology (2144S), Danvers, MA), and HDAC6 (1:100; goat polyclonal; Santa Cruz Biotechnology (sc-5258), Dallas, TX). Membranes were incubated with primary antibody overnight at 4 °C, washed three times with PBST, followed by incubation with horseradish peroxidase-conjugated anti-rabbit (Jackson ImmunoResearch (111–035-144), West Grove, PA), anti-goat (Jackson ImmunoResearch (111–035-144)), or anti-β-actin antibodies (Sigma-Aldrich (A3854)) at room temperature for 2 h. Membranes were then washed three times with PBST. Immunoreactivity for each protein was visualized using a chemiluminescence detection kit (GE Healthcare Life Sciences, Little Chalfont, UK). The images were acquired using ImageQuant LAS 4000 (GE Healthcare Life Sciences) and densitometrically analyzed using ImageJ software.

### Synaptosome isolation and mitochondrial bioenergetics analysis

Synaptosomes were isolated as previously described [11]. Briefly, one hemisphere of the brain was homogenized (10% *w*/*v*) into 0.32 M sucrose solution in HEPES buffer using a glass Dounce homogenizer. The lysate was centrifuged at 1000×*g* for 10 min at 4 °C. The supernatant was mixed with equal volume of 1.3 M sucrose in HEPES buffer and centrifuged at 20,000×*g* for 30 min at 4 °C. The synaptosomal pellet was then resuspended in XF media (Agilent Technologies, Santa Clara, CA) supplemented with 5.5 mM glucose, 0.5 mM sodium pyruvate, and 1 mM glutamine. Oxygen consumption rate (OCR) was measured with an XF24 Flux Analyzer (Agilent Technologies). Oligomycin (6 μM), carbonyl cyanide 4-(trifluoromethoxy)phenylhydrazone (FCCP, 6 μM), and rotenone/antimycin A (2 μM each) (Sigma-Aldrich) were injected sequentially during the assay. An assay cycle of 2-min mix, 3-min wait, and 2-min measure was repeated three times for baseline rates and after each port injection. Basal respiration, ATP-linked respiration, maximal and spare respiratory capacity were determined as described previously [11].

### Transmission electron microscopy

For transmission electron microscopy, synaptosomes were fixed in 2% glutaraldehyde + 2% paraformaldehyde in PBS for over 24 h. Samples were then processed as previously described [11]. The samples were polymerized in a 60 °C oven for approximately 3 days. Ultrathin sections were cut in a Leica Ultracut microtome (Leica Microsystems, Wetzlar, Germany), stained with uranyl acetate and lead citrate in a Leica EM Stainer (Leica Microsystems), and examined in a JEM 1010 transmission electron microscope (JEOL USA, Inc., Peabody, MA) at an accelerating voltage of 80 kV. Digital images were obtained using AMT Imaging System (Advanced Microscopy Techniques Corp, Danvers, MA). 25–35 mitochondria were quantified from each group (4 mice/group, with 5 images/mouse). Atypical mitochondria were identified by 2-fold increases in diameter and/or excessive vacuolization (more than 50% translucent). The percentage of atypical mitochondria was calculated for each group.

### Immunofluorescence analysis

Mice were euthanized by CO2 euthanasia and perfused intracardially with ice-cold PBS. The skin and skull on top of the brain were removed to allow access to the brain. Brains were removed subsequently and transferred to 4% paraformaldehyde. Brains were post-fixed in 4% paraformaldehyde for 24 h, cryoprotected in sucrose, and frozen in optimal cutting temperature compound (Sakura Finetek, Torrance, CA). Coronal brain sections (8 μm) were blocked with blocking buffer (10% normal goat serum, 2% BSA and 0.1% saponin in PBS), followed by incubation with rabbit anti-synaptophysin (1:1000, MilliporeSigma (AB9272), Burlington, MA), rabbit anti-PSD95 (1:1000; Abcam (ab18258)), or mouse anti-phospho-tau (Ser202, Thr205) (AT8) (1:100; ThermoFisher Scientific (MN1020)) diluted in antibody buffer (2% normal goat serum, 2% BSA and 0.1% saponin in PBS) at 4 °C overnight. Slides were then washed three times with PBS, followed by incubation with Alexa-488 goat anti-rabbit (1:1000; Invitrogen (A-21206), Carlsbad, CA) for synaptophysin, Alexa-594 goat anti-rabbit (1:500; Invitrogen (A11037)) for PSD95, and Alexa-488 goat anti-mouse (1:500; Invitrogen (A-21202)) for phospho-tau at room temperature for 2 h. For negative control sections, primary antibody was omitted. After antibody staining, slides were washed three times with PBS, followed by incubation with DAPI (1,5000; Sigma-Aldrich) for 5 min. Slides were then washed three times with PBS, and sealed with FluorSave Reagent (MilliporeSigma). Sections were visualized using a Leica fluorescence microscope (Leica Microsystems). The mean intensity of fluorescence was quantified in three sections (60 μm apart) per mouse using ImageJ software.

### Statistical analysis

Statistical analyses were performed using GraphPad Prism version 7.01 (GraphPad, San Diego, CA). Error bars indicate SEM and statistical significance was assessed by two-way ANOVA with Tukey’s post-hoc analysis. Experimental replicates labeled as “n” were derived from at least four animals as indicated and are described further in each results section. Significance is reported in the results section and full statistical results are detailed in the results section, figure legends and Additional file 1: Table S1.

## Results

### The HDAC6 inhibitor ACY-1083 reverses cisplatin-induced cognitive impairment

Cisplatin (2.3 mg/kg) or PBS was administered intraperitoneally every day for 5 days, followed by 5 days of rest and a second cycle of 5 daily injections. This dosing schedule is commonly used to study neurotoxic effects of cisplatin in mice [11, 31, 33, 35]. The cumulative dose of cisplatin has been shown to effectively control tumor growth in mice [47]. Treatment with the HDAC6 inhibitor ACY-1083 was started 3 days after the last dose of cisplatin and continued for 2 weeks (Fig. 1a). Previous studies of our lab have demonstrated that the cognitive deficits in response to cisplatin are present in both male and female mice with no sex difference observed. For the HDAC6 inhibitors, we have used both male and female mice for our cisplatin-induced peripheral neuropathy study and also did not find any sex difference. Therefore, we used only male mice in the current study.

Behavioral testing to assess cognitive function started 1 week after the last dose of ACY-1083. Mice were first tested in the Y-maze test, which utilizes the mice’s innate tendency to explore a novel environment [15]. Cisplatin-treated mice showed an impaired performance in the Y maze test, as evidenced by a reduction in the rate of perfect alternations (Fig. 1b), implying a decreased spatial working memory function (post-hoc test, *p* = 0.0086). Treatment with 14 doses of the HDAC6 inhibitor ACY-1083 starting 3 days after completion of cisplatin treatment, completely reversed this cognitive deficit (post-hoc test, *p* = 0.0074) (Fig. 1b).

Pharmacokinetic data indicate that ACY-1083, which is an aryl hydroxamate compound, readily penetrates the brain, reaching a brain to plasma ratio of 0.63 at 1-h post injection **(**Table 1**)**. In contrast, the HDAC6 inhibitor ACY-1215, which is an acyl hydroxamate compound, only poorly penetrates the brain (Table 2). Of note, we showed previously that ACY-1083 and ACY-1215 are equally effective in reversing cisplatin-induced peripheral neuropathy [31]. However, treatment with ACY-1215 in the same schedule that reversed peripheral neuropathy did not restore cognitive function **(**Fig. 1c**)** (two-way ANOVA, F (1, 20) = 0.3809, *p* = 0.54). These data indicate that the HDAC6 inhibitor needs to act locally in the brain to reverse cisplatin-induced cognitive deficits. There is no significant difference in the number of total arm entries or the number of entries into each individual arm (two-way ANOVA, F (1, 28) = 0.002633, *p* = 0.9594; F (1, 20) = 0.7648, *p* = 0.3922) (Fig. 1d and e), indicating cisplatin did not affect exploratory behavior in the Y-maze.

**Table 1:** Pharmacokinetic analysis of ACY-1083

		ACY-1083 plasma concentration				ACY-1083 Brain concentration			Brain to plasma ration		
Dose (mg/kg)	Dose route	Sampling time (hr)	Mean (ng/mL)	SD	CV (%)	Mean (ng/g)	SD	CV (%)	Mean	SD	CV (%)
5	IP	Predose	NA	NA	NA	NA	NA	NA	BQL	NA	NA
		0.08	654.33	58.71	8.97	44.13	11.68	26.46	0.07	0.01	18.72
		0.25	936.00	91.43	9.77	109.50	10.26	9.37	0.12	0.01	4.27
		1	180.00	11.00	6.11	112.33	8.08	7.20	0.63	0.08	13.33
		4	78.30	7.39	9.44	15.17	2.41	15.88	0.20	0.04	22.38

Abbreviations: *BQL* below the quantifiable limit of 1.00 ng/mL of ACY-1083 in mouse plasma and brain homogenates, *CV* coefficient of variation, *NA* not available, *SD* standard deviation

**Table 2:** Pharmacokinetic analysis of ACY-1215

		ACY-1215 plasma concentration				ACY-1215 Brain concentration			Brain to plasma ration		
Dose (mg/kg)	Dose route	Sampling time (hr)	Mean (ng/mL)	SD	CV (%)	Mean (ng/g)	SD	CV (%)	Mean	SD	CV (%)
30	Oral	Predose	BQL	N/A	N/A	NA	NA	NA	BQL	NA	NA
		0.5	1226.67	63.51	5.18	15.20	3.65	24.01	0.01	0.00	27.61
		1	561.00	128.36	22.88	5.61	NA	NA	NA	NA	NA
		4	60.60	26.63	43.94	BQL	NA	NA	NA	NA	NA
		8	18.02	9.78	54.29	BQL	NA	NA	NA	NA	NA

Abbreviations: *BQL* below the quantifiable limit of 1.00 ng/mL of ACY-1215 in mouse plasma and brain homogenates, *CV* coefficient of variation, *NA* not available, *SD* standard deviation

**Fig. 2:**
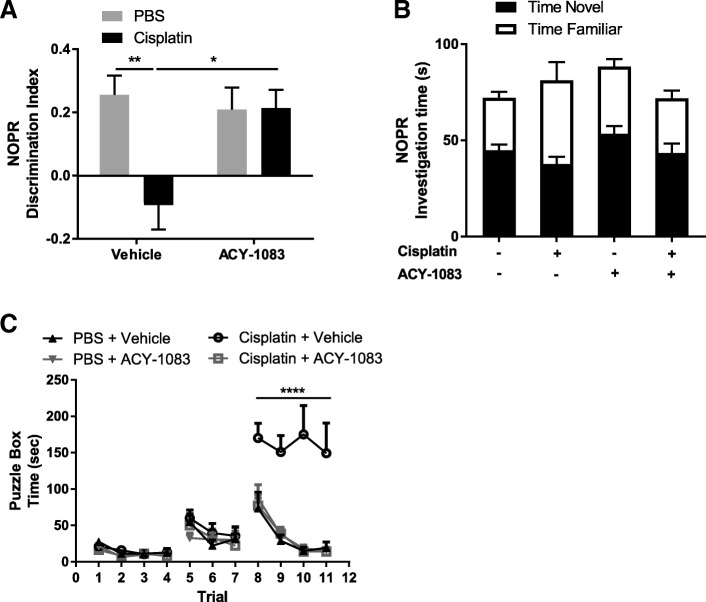
Effect of the brain-penetrating HDAC6 inhibitor ACY-1083 on cisplatin-induced cognitive impairment in the NOPR and puzzle box tests**.** Mice were treated with two 5-day cycles of cisplatin or PBS, followed by 14 daily administrations of ACY-1083. **a** The NOPR test was performed 1 week after the last injection of ACY-1083 and the discrimination index was calculated (*n* = 8–9; two-way ANOVA with Tukey’s post-hoc analysis: F (1, 30) = 6.3; PBS vs. Cisplatin, *p* = 0.0076; Cisplatin vs. Cisplatin + ACY-1083, *p* = 0.0223). **b** Total investigation time of both objects in the NOPR was recorded (*n* = 8–9; two-way ANOVA, (F (1, 31) = 0.6425, *p* = 0.4289), stacked columns were used to indicate time of interaction with the novel and the familiar object. **c** The puzzle box was performed 2 weeks after the last injection of ACY-1083. During trials 1–4, the underpass was unobstructed; during trials 5–7, the underpass was filled with corncob bedding; during trials 8–11, the underpass was covered by the cardboard plug. The time it took for mice to enter the goal box was recorded (*n* = 10–14; two-way ANOVA with Tukey’s post-hoc analysis: F (30, 407) = 5.698; PBS vs. Cisplatin, *p* < 0.0001; Cisplatin vs. Cisplatin + ACY-1083, *p* < 0.0001). Results are expressed as mean ± SEM; *p < 0.05, **p < 0.01, ****p < 0.0001

To further explore the capacity of ACY-1083 to reverse cisplatin-induced cognitive impairment, additional behavioral tests including the novel object/place recognition (NOPR) test [3] and the puzzle box test [5] were used. The NOPR test assesses spatial working memory in mice based on their innate preference for novelty [3]. In this test, control mice showed a clear preference for the novel object, whereas cisplatin-treated mice lost this preference (post-hoc test, *p* = 0.0076) (Fig. 2a) [11, 54]. The loss of preference for the novel object/place was fully reversed by the 2-week regimen of ACY-1083 treatment (post-hoc test, *p* = 0.0223) **(**Fig. 2a**)**. No differences were detected in the total interaction time with objects (two-way ANOVA, F (1, 31) = 0.6425, *p* = 0.4289) (Fig. 2b), indicating that cisplatin and ACY-1083 had no effect on locomotor activity or interest in the objects.

The puzzle box test has been established as a problem-solving task to study executive functioning in mice [5]. During the test, mice were introduced into the brightly lit start zone, and the time to enter the dark goal box via a connecting underpass was recorded. There was no effect of cisplatin during trial 1–4 when the underpass was unobstructed, or trial 5–7 when the underpass was filled with bedding. However, cisplatin-treated mice used significantly more time than the PBS-treated mice when the difficult obstacle (cardboard plug, trials 8–11) was covering the underpass (post-hoc test, *p* < 0.0001) (Fig. 2c). Notably, 2 weeks of ACY-1083 treatment completely restored the executive functioning of cisplatin-treated mice (post-hoc test, p < 0.0001). ACY-1083 alone did not have any effect in any of the behavioral tests.

Taken together, these findings indicate that cisplatin impaired spatial working memory, executive functioning, and problem-solving abilities in mice and, more importantly, that the brain-penetrating HDAC6 inhibitor ACY-1083 reversed these deficits.

### ACY-1083 modulates α-tubulin acetylation in the brain

**Fig. 3:**
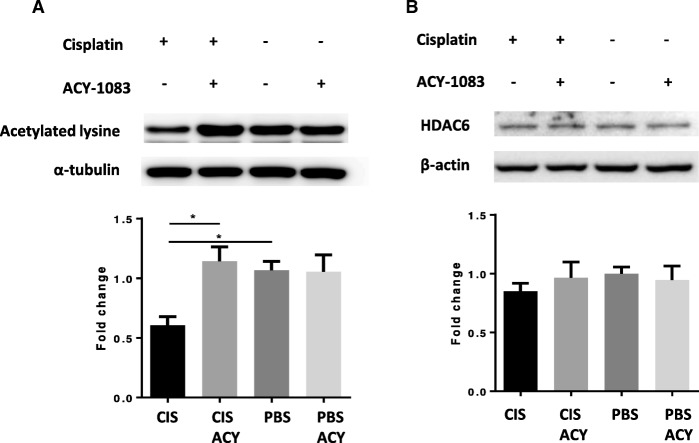
Effect of cisplatin and ACY-1083 on α-tubulin acetylation and HDAC6 expression. Mice were treated with two 5-day cycles of cisplatin or PBS, followed by 11 daily administrations of ACY-1083. Brains were collected 3 h after the last injection of ACY-1083. **a** Acetylated α-tubulin (*n* = 4; two-way ANOVA with Tukey’s post-hoc analysis: F (1, 12) = 6.765; PBS vs. Cisplatin, *p* = 0.0408; Cisplatin vs. Cisplatin + ACY-1083, *p* = 0.0170) and (**b**) HDAC6 expression (*n* = 4; two-way ANOVA, F (1, 12) = 0.7129, *p* = 0.4150) levels were assessed by Western blot analysis. Results are expressed as mean ± SEM; *P < 0.05

Given that α-tubulin is a major target of HDAC6 [25], we assessed the effects of cisplatin and ACY-1083 on α-tubulin acetylation in the brain. Figure 3a shows that cisplatin treatment decreased α-tubulin acetylation in the brain (post-hoc test, *p* = 0.0408) and that this was reversed by ACY-1083 treatment (post-hoc test, *p* = 0.0170). To determine if the cisplatin-induced decrease in α-tubulin acetylation was due to changes in HDAC6 protein levels, we also probed for HDAC6. As shown in Fig. 3b, cisplatin did not change the protein level of HDAC6 in the brain (two-way ANOVA, F (1, 12) = 0.7129, *p* = 0.4150).

### ACY-1083 reverses cisplatin-induced impairment in synaptosomal mitochondrial morphology and bioenergetics

**Fig. 4:**
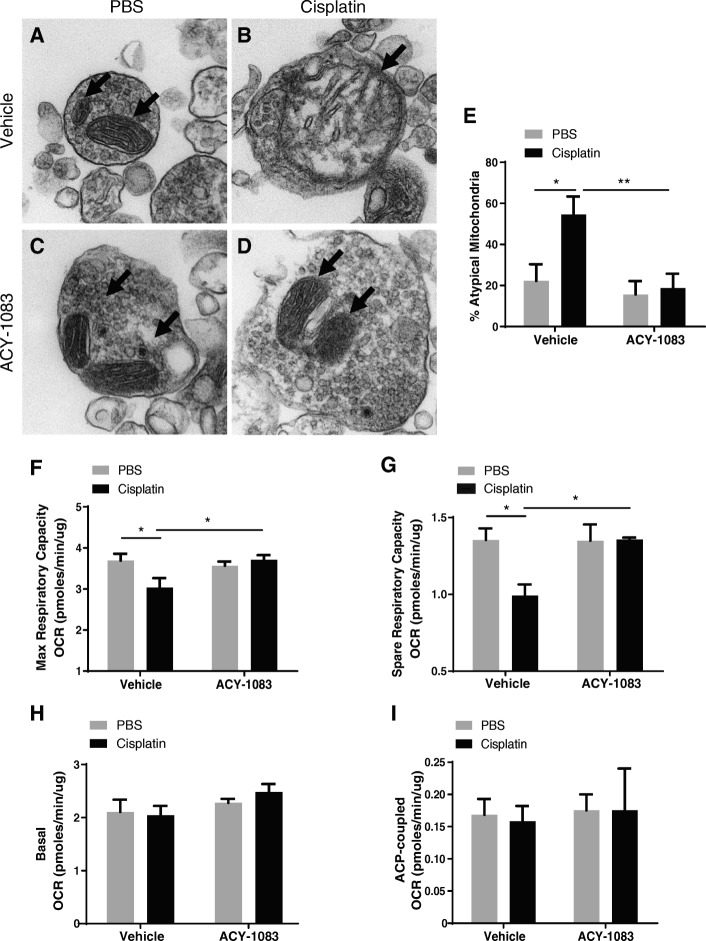
Effect of cisplatin and ACY-1083 on synaptosomal mitochondrial deficits. After completion of all behavioral tests, synaptosomes were isolated from brains of mice treated with (**a**) PBS + vehicle, (**b**) cisplatin + vehicle, (**c**) PBS + ACY-1083, or (**d**) cisplatin + ACY-1083. Mitochondrial morphology was assessed on electron microscopic images. Mitochondria were indicated by arrows. **e** Percentage of atypical mitochondria was quantified (*n* = 4 mice (25–35 mitochondria) per group; two-way ANOVA with Tukey’s post-hoc analysis: F (1, 120) = 7.57; PBS vs. Cisplatin, *p* = 0.0256; Cisplatin vs. Cisplatin + ACY-1083, *p* = 0.0060). Oxygen consumption rates were analyzed in isolated synaptosomes using the Seahorse XFe24 Flux Analyzer. **f** Maximum respiratory capacity (MRC) (*n* = 6; two-way ANOVA with Tukey’s post-hoc analysis: F (1, 20) = 6.243; PBS vs. Cisplatin, *p* = 0.0416; Cisplatin vs. Cisplatin + ACY-1083, *p* = 0.0345), (**g**) spare respiratory capacity (SRC) (*n* = 6; two-way ANOVA with Tukey’s post-hoc analysis: F (1, 20) = 5.296; PBS vs. Cisplatin, *p* = 0.0147; Cisplatin vs. Cisplatin + ACY-1083, *p* = 0.0291; *n* = 6), (**h**) basal respiration (*n* = 6; two-way ANOVA, F (1, 20) = 0.6219, *p* = 0.4396), and (**i**) ATP-coupled respiration (*n* = 6; two-way ANOVA, F (1, 20) = 0.02046, *p* = 0.8877) were calculated. Results are expressed as means ± SEM; *p < 0.05, **p < 0.01

Synaptosomal mitochondrial dysfunction is a key player in cisplatin-induced cognitive impairment [11]. Pharmacological or genetic manipulation of HDAC6 has been shown to enhance neuronal mitochondrial transport [10, 31, 34]. Therefore, we tested whether the alterations in synaptosomal mitochondrial morphology and function as a result of cisplatin were reversed by ACY-1083 treatment. As shown in Figs. 4a–e, cisplatin treatment led to morphological damage to synaptosomal mitochondria, as evidenced by an increased percentage of atypical mitochondria that were swollen and/or had abnormal cristae structure (post-hoc test, *p* = 0.0256). Notably, these morphological changes were completely reversed by 2 weeks of ACY-1083 treatment (post-hoc test, *p* = 0.0060).

Furthermore, cisplatin induced significant decreases in parameters measuring synaptosomal mitochondrial bioenergetics, including the maximal respiratory capacity (MRC) (post-hoc test, *p* = 0.0416) (Fig. 4f) and spare respiratory capacity (SRC) (post-hoc test, *p* = 0.0147) (Fig. 4g). ACY-1083 treatment also restored cisplatin-induced impairment in MRC (post-hoc test, *p* = 0.0345) and SRC (post-hoc test, *p* = 0.0291) (Fig. 4f and g). Cisplatin and ACY-1083 did not affect basal respiration (two-way ANOVA, F (1, 20) = 0.6219, *p* = 0.4396) (Fig. 4h) or ATP-coupled respiration (two-way ANOVA, F (1, 20) = 0.02046, *p* = 0.8877; *n* = 6) (Fig. 4i).

### ACY-1083 restores expression of markers of synaptic integrity in cisplatin-treated mice

**Fig. 5:**
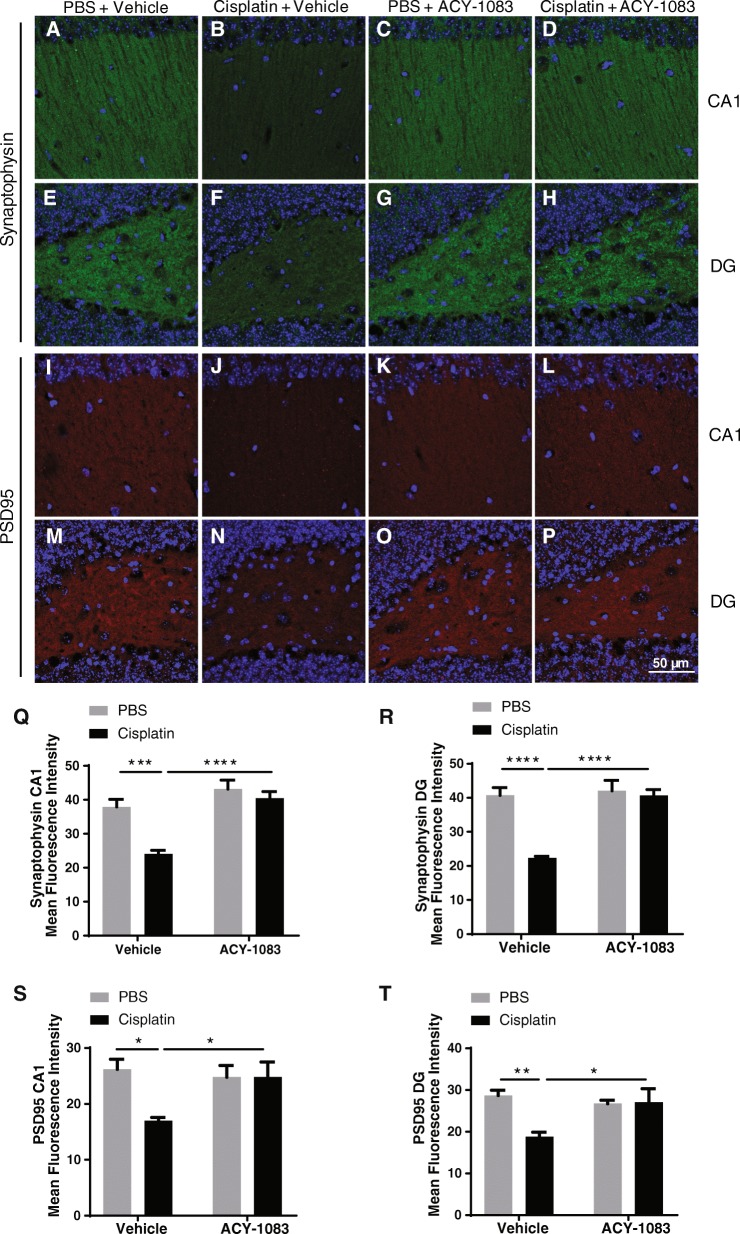
Effect of cisplatin and ACY-1083 on markers of synaptic integrity. Brains were collected after completion of all behavioral tests and processed for immunofluorescence analysis of synaptophysin and PSD95 in the CA1 and DG regions of the hippocampus. Mean fluorescence intensity was quantified. **a**–**d**, **q** Example images and quantification for synaptophysin staining in CA1 region from mice treated with (**a**) PBS + vehicle, (**b**) cisplatin + vehicle, (**c**) PBS + ACY-1083, or (**d**) cisplatin + ACY-1083 (*n* = 7; two-way ANOVA with Tukey’s post-hoc analysis: F (1, 24) = 7.169; PBS vs. Cisplatin, *p* = 0.0005; Cisplatin vs. Cisplatin + ACY-1083, *p* < 0.0001). **e**–**h**, **r** Example images and quantification for synaptophysin staining in DG region from mice treated with (**e**) PBS + vehicle, (**f**) cisplatin + vehicle, **g** PBS + ACY-1083, or (**h**) cisplatin + ACY-1083 (*n* = 7; two-way ANOVA with Tukey’s post-hoc analysis: F (1, 24) = 15.97; PBS vs. Cisplatin, *p* < 0.0001; Cisplatin vs. Cisplatin + ACY-1083, *p* < 0.0001). **i**–**l**, **s** Example images and quantification for PSD95 staining in CA1 region from mice treated with (**i**) PBS + vehicle, (**j**) cisplatin + vehicle, (**k**) PBS + ACY-1083, or (**l**) cisplatin + ACY-1083 (*n* = 7; two-way ANOVA with Tukey’s post-hoc analysis: F (1, 24) = 5.554; PBS vs. Cisplatin, *p* = 0.0138; Cisplatin vs. Cisplatin + ACY-1083, *p* = 0.0434). **m**–**p**, **t** Example images and quantification for PSD95 staining in DG region from mice treated with (**m**) PBS + vehicle, (**n**) cisplatin + vehicle, (**o**) PBS + ACY-1083, or (**p**) cisplatin + ACY-1083 (*n* = 7; two-way ANOVA with Tukey’s post-hoc analysis: F (1, 24) = 7.052; PBS vs. Cisplatin, *p* = 0.0065; Cisplatin vs. Cisplatin + ACY-1083, *p* = 0.0275). Results are expressed as means ± SEM; *P < 0.05, **P < 0.01

Synaptic integrity enables effective synaptic transmission, and is therefore crucial for memory formation and learning. Synaptic dysfunction is one of the early pathological features of cognitive decline in dementia in both human and animal models [6]. Therefore, we examined the effect of cisplatin and ACY-1083 on the expression level of markers of synaptic integrity in the hippocampal cornu ammonis 1 (CA1) and dentate gyrus (DG) regions. The results in Figs. 5 demonstrate that cisplatin reduced the expression of the presynaptic marker synaptophysin (post-hoc, *p* = 0.0005 for CA1, *p* < 0.0001 for DG) and the postsynaptic marker PSD95 (post-hoc, *p* = 0.0138 for CA1, *p* = 0.0065 for DG) in the CA1 and DG regions of the hippocampus. Two weeks of ACY-1083 treatment normalized expression of both the presynaptic (post-hoc, *p* < 0.0001 for CA1, *p* < 0.0001 for DG) and postsynaptic markers (post-hoc, *p* = 0.0434 for CA1, *p* = 0.0275 for DG).

### ACY-1083 reverses tau pathology in cisplatin-treated mice

**Fig. 6:**
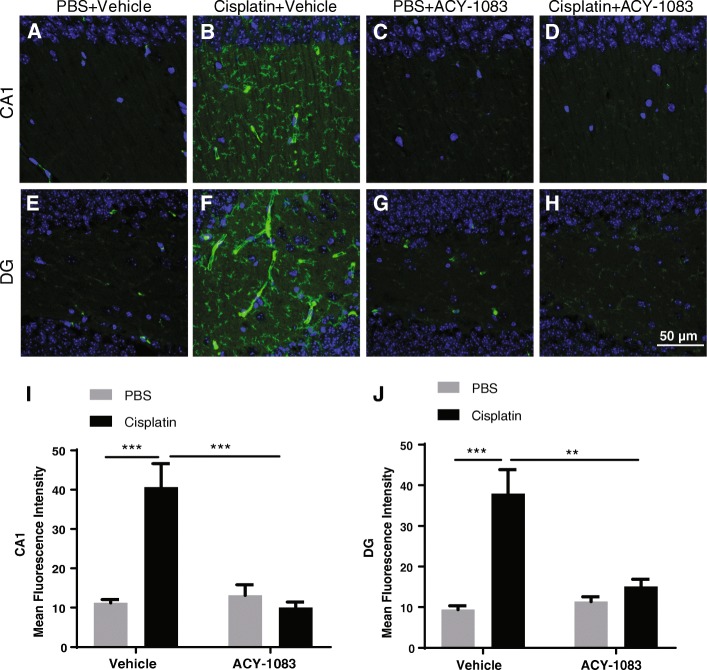
Effect of cisplatin and ACY-1083 on tau phosphorylation. Brains were collected after completion of behavioral tests and processed for immunofluorescence analysis of p-tau in the CA1 and DG regions. Mean fluorescence intensity was quantified. **a**–**d**, **i** Example images and quantification for p-tau staining in CA1 region from mice treated with (**a**) PBS + vehicle, (**b**) cisplatin + vehicle, (**c**) PBS + ACY-1083, or (**d**) cisplatin + ACY-1083 (n = 4; two-way ANOVA with Tukey’s post-hoc analysis: F(1, 12) = 22.85; PBS vs. Cisplatin, *p* = 0.0003; Cisplatin vs. Cisplatin + ACY-1083, *p* = 0.0002). **e**–**h**, **j** Example images and quantification for p-tau staining in DG region from mice treated with (**e**) PBS + vehicle, (**f**) cisplatin + vehicle, (**g**) PBS + ACY-1083, or (**h**) cisplatin + ACY-1083 (n = 4; two-way ANOVA with Tukey’s post-hoc analysis: F (1, 12) = 15.38; PBS vs. Cisplatin, p = 0.0002; Cisplatin vs. Cisplatin + ACY-1083, *p* = 0.0013). Results are expressed as means ± SEM; **P < 0.01, ***P < 0.001

Dysregulation of tau has been implicated as a fundamental contributor to cognitive decline in neurodegenerative diseases [29]. Accumulation of hyperphosphorylated tau correlates with onset of cognitive decline in animal models of AD [19, 38]. Moreover, tau pathology is closely associated with dysregulation and loss of synaptic connections [37, 53]. We therefore examined if cisplatin-induced cognitive impairment and synaptic dysregulation is associated with pathological tau. We detected significant upregulation of tau phosphorylation with the AT8 antibody in the CA1 (post-hoc, *p* = 0.0003) (Figs. 6a–d) and DG (post-hoc, *p* = 0.0002) (Figs. 6e–h) regions of cisplatin-treated mice. Notably, ACY-1083 treatment completely reversed tau hyperphosphorylation induced by cisplatin treatment (post-hoc, *p* = 0.0002 for CA1, *p* = 0.0013 for DG) (Fig. 6i and j).

## Discussion

We show here for the first time that pharmacological inhibition of HDAC6 with the brain-penetrating inhibitor ACY-1083 reverses cisplatin-induced cognitive impairment as assessed in multiple behavioral tests. We have previously demonstrated that the same regimen of HDAC6 inhibition resolves cisplatin-induced neuropathy [31]. Moreover, HDAC6 inhibitors have been shown to enhance tumor control in Phase I and II clinical trials [46]. Therefore, inhibition of HDAC6 appears as a promising therapeutic strategy for reversing neurotoxic side effects of chemotherapy while improving the efficacy of cancer treatment.

Our result suggests that penetration of the inhibitor into the brain is likely required, as the non-brain-penetrating HDAC6 inhibitor ACY-1215 had no effect on cognitive function at a dosing schedule that reversed cisplatin-induced peripheral neuropathy [31]. The reversal of cisplatin-induced cognitive impairment by the brain-penetrating inhibitor ACY-1083 was associated with reversal of synaptosomal mitochondrial deficits and restoration of synaptic integrity. Furthermore, we demonstrate that deacetylation of α-tubulin and hyperphosphorylation of tau were part of the mechanism contributing to cisplatin-induced cognitive impairment, and that HDAC6 inhibition reversed these pathological changes as well.

Cognitive impairment in neurodegenerative diseases is frequently accompanied by disruption of microtubule stability [4, 41]. However, no such findings have ever been reported in CICI. To our knowledge, we are the first to demonstrate that cisplatin-induced cognitive impairment is associated with deacetylation of the microtubule protein α-tubulin and hyperphosphorylation of the microtubule-associated protein tau, which are indicative of changes in microtubule stability. Our data indicate that cisplatin induced a significant reduction in α-tubulin acetylation in the brain. We propose that this could be attributed to increased HDAC6 activity, as no change in HDAC6 protein level was detected. However, downregulation or inhibition of α-tubulin acetyltransferases, such as αTAT1, could also be involved in cisplatin-induced α-tubulin deacetylation [27]. Inhibition of HDAC6 normalized the level of acetylated α-tubulin in the brain. ACY-1083 alone did not have any effect. This is consistent with a previous report showing no detectable changes in α-tubulin acetylation in brains of HDAC6 knockout mice, which was attributed to high baseline level of α-tubulin acetylation in the brain [52].

The microtubule-associated protein tau plays an essential role in the modulation of microtubule assembly and stability and the maintenance of normal neuronal morphology. Dysregulation of tau, including tau hyperphosphorylation and formation of neurofilament tangles, has been reported in various experimental models of aging and neurodegenerative diseases, as well as in AD patients [29]. Recently, tau pathology has also been linked to cognitive disorders that develop in response to head trauma, where perivascular accumulation of abnormally phosphorylated tau was detected in the depths of cortical sulci [42]. We show here that cisplatin induced tau hyperphosphorylation in the DG and CA1 regions of the hippocampus, a phenomenon that correlate with onset of cognitive decline in AD and other tauopathy models [19, 38], and that ACY-1083 treatment reversed tau hyperphosphorylation.

Hyperphosphorylated tau displays reduced binding to microtubules, leading to disrupted microtubule assembly, hyperdynamic microtubules, and decreased axonal transport of cellular organelles, including the mitochondria [41]. More specifically, pathological phosphorylation of tau has been shown to inhibit kinesin-1 motility, thereby limiting kinesin-1-dependent axonal mitochondrial transport [41]. In contrast, increases in α-tubulin acetylation can enhance mitochondrial movement through increased recruitment of kinesin-1 to microtubules [10]. In our cisplatin model of CICI, we observed morphologically distorted and functionally disrupted mitochondria in the synaptosomes. Importantly, these cisplatin-induced synaptosomal mitochondrial deficits were reversed by the HDAC6 inhibitor ACY-1083, along with restoration of α-tubulin acetylation and reversal of tau hyperphosphorylation. We have found evidence for disruption of mitochondrial trafficking by cisplatin in sensory neurons, which was rescued by selective inhibition of HDAC6 [31]. Therefore, we propose the normalization of α-tubulin acetylation and tau phosphorylation as mechanism of action for HDAC6 inhibitors, which allows stabilization of microtubules and restoration of microtubule-dependent axonal transport of mitochondria, thereby restoring synaptosomal mitochondrial function. In this respect, data showing that pharmacological or genetic disruption of HDAC6 is protective against pathological tau-induced microtubule abnormality and cognitive decline are of great interest [9, 13, 20, 34, 39, 50]. For example, Xiong et al. demonstrated that HDAC6 null mutation or HDAC6 inhibition rescued tau-induced microtubule defects and neuromuscular junction abnormalities in drosophila by enhancing microtubule acetylation [50]. The HDAC6 inhibitor tubastatin has been shown to reduce the level of total tau and restore memory function in the rTg4510 mouse model of AD [39]. Taken together, we propose that HDAC6 inhibition restores cognitive function in cisplatin-treated mice through reversal of tau phosphorylation and normalization of α-tubulin acetylation, leading to restoration of synaptosomal mitochondrial function.

Synaptic dysfunction and loss of synaptic integrity are among the early signs of neurodegeneration [6]. In our previous studies, we have shown that cisplatin decreased dendritic spine density and dendritic arborization in the cingulate cortex [54], which reflects alterations in the functionality of resident synapses [7]. Following this line of thought, we observed here disrupted synaptic integrity in response to cisplatin, as evidenced by decreases in presynaptic and postsynaptic markers in the hippocampus. Moreover, we show that ACY-1083 restored synaptic integrity and that this corresponded with reversal of behavioral deficits. In neurons, synapses represent sites of high energy demand and extensive Ca^2+^ fluctuation, where large amounts of mitochondria are needed to maintain proper synaptic function and integrity [40]. These features render synapses extremely susceptible to perturbations in mitochondrial function and trafficking [8]. Pathological tau has also been linked to synaptotoxicity [37]. Intracellular accumulation of tau induces synaptic degeneration and memory deficits [51]. In AD brains, the presence of hyperphosphorylated tau highly correlates with reduction in presynaptic protein expression [12]. Pathogenic tau binds to synaptic vesicles and interferes with presynaptic functions by lowering synaptic vesicle mobility and release rate [53]. Given the protective effects of ACY-1083 on synaptosomal mitochondrial function and tau pathology, we propose that HDAC6 inhibition restores synaptic integrity at least partially through normalization of mitochondrial transport and function, and reversal of tau hyperphosphorylation.

In the current study, we show that cisplatin induced a significant decrease in the acetylation of α-tubulin without affecting the expression level of HDAC6, which suggests that cisplatin may enhance the deacetylase activity of HDAC6. Previous studies have implicated tau as a deacetylation target of HDAC6 [9, 43, 44]. Therefore, we examined tau acetylation with the only commercially available antibody that recognizes tau acetylation at the lysine residue K280, a previously identified target site for HDAC6 [43, 44]. However, we did not detect any change associated with tau acetylation at K280 with either cisplatin or ACY-1083 (data not shown). We cannot rule out the possibility that cisplatin and/or ACY-1083 modulate tau phosphorylation through regulation of acetylation on other lysine residues, such as the KXGS motifs within the microtubule-binding domain. Of note, tau hypoacetylation on KXGS motifs has been associated with tau hyperphosphorylation and cognitive deficits in a mouse model of tauopathy and in AD patients [13]. Furthermore, the tau hyperphosphorylation phenotype observed in mice with tauopathy can be reversed by HDAC6 inhibition-mediated tau acetylation [13]. Therefore, the tau pathology observed in our cisplatin model might be attributed to upregulated HDAC6 activity leading to hypoacetylation of tau on certain residues, which was reversed by ACY-1083. The deacetylase activity of HDAC6 can be regulated by its posttranslational modification status [10, 32, 48]. For example, upregulated deacetylase activity of HDAC6 has been associated with its phosphorylation by GSK3β, GRK2, and CK2 [10, 32, 48]. Interestingly, activation of some of these kinases, such as GSK3β and CK2, are implicated in neurodegeneration and tau hyperphosphorylation [19]. Therefore, it is possible that activation of one or more of these protein kinases underlies cisplatin-induced tau hyperphosphorylation, HDAC6 activation and α-tubulin deacetylation.

## Conclusions

In summary, our results provide evidence that the brain-penetrating HDAC6 inhibitor ACY-1083 is a promising therapeutic candidate for the treatment of CICI. Our findings indicate that dysregulation of microtubule dynamics, including deacetylation of α-tubulin and hyperphosphorylation of tau, contribute to the pathogenesis of CICI. Inhibition of HDAC6 reverses cisplatin-induced α-tubulin deacetylation and tau hyperphosphorylation and restores synaptosomal mitochondrial function, which likely contribute to the restoration of synaptic integrity. Importantly, HDAC6 inhibitors have been shown to be safe and active as add-on cancer therapy [46]. Therefore, they hold great promises as a class of drugs that could reverse chemotherapy-induced neurotoxicity without negatively affecting cancer treatment.

## Additional file

Additional file 1: **Table S1.** Statistical analyses. (DOCX 25 kb)
