# Supplementary material for: Pharmacological inhibition of HDAC6 reverses cognitive impairment and tau pathology as a result of cisplatin treatment
Source: Acta Neuropathol Commun. 2018 Oct 1;6:103. doi: 10.1186/s40478-018-0604-3 (PMC6166273; doi:10.1186/s40478-018-0604-3)
Supplement: Supplementary file 1 — Table S1. Statistical analyses. (DOCX 25 kb) [file 40478_2018_604_MOESM1_ESM.docx]

**Table S1. Statistical analyses**

| **Figure** | **Comparison** | **Statistical Test** | **P value** | **Confidence interval** |
| --- | --- | --- | --- | --- |
| Fig. 1B | PBS vs. Cisplatin; Vehicle vs. ACY-1083 | Two-way ANOVA | Interaction: p = 0.0258; vehicle vs. ACY-1083: p= 0.0132; PBS vs. Cisplatin: p = 0.0160 |  |
| Fig. 1B | Vehicle:PBS vs. Vehicle:Cisplatin | Tukey's post hoc | 0.0086 | 2.417, 20.08 |
| Fig. 1B | Vehicle:Cisplatin vs. ACY-1083:PBS | Tukey's post hoc | 0.0051 | -20.75, -3.08 |
| Fig. 1B | Vehicle:Cisplatin vs. ACY-1083:Cisplatin | Tukey's post hoc | 0.0074 | -20.27, -2.605 |
| Fig. 1C | PBS vs. Cisplatin; Vehicle vs. ACY-1215 | Two-way ANOVA | Interaction: p = 0.5441; vehicle vs. ACY-1083: p= 0.7270; PBS vs. Cisplatin: p < 0.0001 |  |
| Fig. 1C | Vehicle:PBS vs. Vehicle:Cisplatin | Tukey's post hoc | 0.0122 | 2.959, 28.02 |
| Fig. 1C | Vehicle:PBS vs. ACY-1215:Cisplatin | Tukey's post hoc | 0.0080 | 3.793, -28.86 |
| Fig. 1C | Vehicle:Cisplatin vs. ACY-1215:PBS | Tukey's post hoc | 0.0026 | -31.1, -6.034 |
| Fig. 1C | ACY-1215:PBS vs. ACY-1215:Cisplatin | Tukey's post hoc | 0.0017 | -6.868, 31.93 |
| Fig. 1D | ACY-1083:PBS vs. ACY-1083:Cisplatin | Two-way ANOVA | Interaction: p = 0.9594; vehicle vs. ACY-1083: p= 0.1015; PBS vs. Cisplatin: p = 0.1479 |  |
| Fig. 1E | ACY-1215:PBS vs. ACY-1215:Cisplatin | Two-way ANOVA | Interaction: p = 0.3922; vehicle vs. ACY-1215: p= 0.8629; PBS vs. Cisplatin: p = 0.1950 |  |
| Fig. 2A | ACY-1083:PBS vs. ACY-1083:Cisplatin | Two-way ANOVA | Interaction: p = 0.0177; vehicle vs. ACY-1215: p= 0.0773; PBS vs. Cisplatin: p = 0.0208 |  |
| Fig. 2A | Vehicle:PBS vs. Vehicle:Cisplatin | Tukey's post hoc | 0.0076 | 0.07584, 0.6036 |
| Fig. 2A | Vehicle:Cisplatin vs. ACY-1083:PBS | Tukey's post hoc | 0.0306 | -0.5645, -0.02149 |
| Fig. 2A | Vehicle:Cisplatin vs. ACY-1083:Cisplatin | Tukey's post hoc | 0.0223 | -0.5616, -0.03389 |
| Fig. 2B | ACY-1083:PBS vs. ACY-1083:Cisplatin | Two-way ANOVA | Interaction: p = 0.4289; vehicle vs. ACY-1083: p= 0.1768; PBS vs. Cisplatin: p = 0.6244 |  |
| Fig. 2C | ACY-1083:PBS vs. ACY-1083:Cisplatin | Two-way repeated measures ANOVA | Interaction: p < 0.0001; vehicle vs. ACY-1083: p < 0.0001; PBS vs. Cisplatin: p < 0.0001 |  |
| Fig. 2C | Trial 8 Vehicle:PBS vs. Vehicle:Cisplatin | Tukey's post hoc | < 0.0001 | 56.51, 136.8 |
| Fig. 2C | Trial 8 Vehicle:Cisplatin vs. ACY-1083:PBS | Tukey's post hoc | < 0.0001 | 54.76, 131 |
| Fig. 2C | Trial 8 Vehicle:Cisplatin vs. ACY-1083:Cisplatin | Tukey's post hoc | < 0.0001 | 43.54, 121.7 |
| Fig. 2C | Trial 9 Vehicle:PBS vs. Vehicle:Cisplatin | Tukey's post hoc | < 0.0001 | 81.96, 162.2 |
| Fig. 2C | Trial 9 Vehicle:Cisplatin vs. ACY-1083:PBS | Tukey's post hoc | < 0.0001 | 74.2, 150.5 |
| Fig. 2C | Trial 9 Vehicle:Cisplatin vs. ACY-1083:Cisplatin | Tukey's post hoc | < 0.0001 | 74.49, 152.6 |
| Fig. 2C | Trial 10 Vehicle:PBS vs. Vehicle:Cisplatin | Tukey's post hoc | < 0.0001 | 89.41, 231 |
| Fig. 2C | Trial 10 Vehicle:Cisplatin vs. ACY-1083:PBS | Tukey's post hoc | < 0.0001 | 90.41, 232 |
| Fig. 2C | Trial 10 Vehicle:Cisplatin vs. ACY-1083:Cisplatin | Tukey's post hoc | < 0.0001 | 86.41, 228 |
| Fig. 2C | Trial 11 Vehicle:PBS vs. Vehicle:Cisplatin | Tukey's post hoc | < 0.0001 | 59.07, 200.7 |
| Fig. 2C | Trial 11 Vehicle:Cisplatin vs. ACY-1083:PBS | Tukey's post hoc | < 0.0001 | 64.41, 206 |
| Fig. 2C | Trial 11 Vehicle:Cisplatin vs. ACY-1083:Cisplatin | Tukey's post hoc | < 0.0001 | 62.07, 203.7 |
| Fig. 3A | ACY-1083:PBS vs. ACY-1083:Cisplatin | Two-way ANOVA | Interaction: p = 0.0232; vehicle vs. ACY-1083: p = 0.0295; PBS vs. Cisplatin: p = 0.1033 |  |
| Fig. 3A | Vehicle:PBS vs. Vehicle:Cisplatin | Tukey's post hoc | 0.0408 | 0.01749, 0.9059 |
| Fig. 3A | Vehicle:Cisplatin vs. ACY-1083:PBS | Tukey's post hoc | 0.0479 | -0.8921, -0.003682 |
| Fig. 3A | Vehicle:Cisplatin vs. ACY-1083:Cisplatin | Tukey's post hoc | 0.0170 | -0.9808, -0.09233 |
| Fig. 3B | ACY-1083:PBS vs. ACY-1083:Cisplatin | Two-way ANOVA | Interaction: p = 0.4150; vehicle vs. ACY-1083: p = 0.7742; PBS vs. Cisplatin: p = 0.5262 |  |
| Fig. 4E | ACY-1083:PBS vs. ACY-1083:Cisplatin | Two-way ANOVA | Interaction: p = 0.045; vehicle vs. ACY-1083: p = 0.0069; PBS vs. Cisplatin: p = 0.0231 |  |
| Fig. 4E | Vehicle:PBS vs. Vehicle:Cisplatin | Tukey's post hoc | 0.0256 | -62.09, -2.555 |
| Fig. 4E | Vehicle:Cisplatin vs. ACY-1083:PBS | Tukey's post hoc | 0.0022 | 10.46 to 67.38 |
| Fig. 4E | Vehicle:Cisplatin vs. ACY-1083:Cisplatin | Tukey's post hoc | 0.0060 | 7.335 to 64.26 |
| Fig. 4F | ACY-1083:PBS vs. ACY-1083:Cisplatin | Two-way ANOVA | Interaction: p = 0.0213; vehicle vs. ACY-1083: p = 0.1021; PBS vs. Cisplatin: p = 0.1283 |  |
| Fig. 4F | Vehicle:PBS vs. Vehicle:Cisplatin | Tukey's post hoc | 0.0416 | 0.02043, 1.297 |
| Fig. 4F | Trial 8 Vehicle:Cisplatin vs. ACY-1083:Cisplatin | Tukey's post hoc | 0.0345 | -1.318, -0.04088 |
| Fig. 4G | ACY-1083:PBS vs. ACY-1083:Cisplatin | Two-way ANOVA | Interaction: p = 0.0343; vehicle vs. ACY-1083: p = 0.0385; PBS vs. Cisplatin: p = 0.0419 |  |
| Fig. 4G | Vehicle:PBS vs. Vehicle:Cisplatin | Tukey's post hoc | 0.0147 | 0.06358, 0.6585 |
| Fig. 4G | Vehicle:Cisplatin vs. ACY-1083:PBS | Tukey's post hoc | 0.0223 | -0.6683, -0.04439 |
| Fig. 4G | Vehicle:Cisplatin vs. ACY-1083:Cisplatin | Tukey's post hoc | 0.0291 | -0.697, -0.03192 |
| Fig. 4H | ACY-1083:PBS vs. ACY-1083:Cisplatin | Two-way ANOVA | Interaction: p = 0.4396; vehicle vs. ACY-1083: p = 0.0852; PBS vs. Cisplatin: p = 0.6777 |  |
| Fig. 4I | ACY-1083:PBS vs. ACY-1083:Cisplatin | Two-way ANOVA | Interaction: p = 0.8877; vehicle vs. ACY-1083: p = 0.7416; PBS vs. Cisplatin: p = 0.8852 |  |
| Fig. 5Q | ACY-1083:PBS vs. ACY-1083:Cisplatin | Two-way ANOVA | Interaction: p = 0.0132; vehicle vs. ACY-1083: p < 0.0001; PBS vs. Cisplatin: p = 0.0006 |  |
| Fig. 5Q | Vehicle:PBS vs. Vehicle:Cisplatin | Tukey's post hoc | 0.0005 | 5.744, 22.01 |
| Fig. 5Q | Vehicle:Cisplatin vs. ACY-1083:PBS | Tukey's post hoc | < 0.0001 | -27.27, -11 |
| Fig. 5Q | Vehicle:Cisplatin vs. ACY-1083:Cisplatin | Tukey's post hoc | < 0.0001 | -24.55, -8.287 |
| Fig. 5R | ACY-1083:PBS vs. ACY-1083:Cisplatin | Two-way ANOVA | Interaction: p = 0.0005; vehicle vs. ACY-1083: p = 0.0001; PBS vs. Cisplatin: p = 0.0001 |  |
| Fig. 5R | Vehicle:PBS vs. Vehicle:Cisplatin | Tukey's post hoc | < 0.0001 | 10.07, 26.64 |
| Fig. 5R | Vehicle:Cisplatin vs. ACY-1083:PBS | Tukey's post hoc | < 0.0001 | -28, -11.43 |
| Fig. 5R | Vehicle:Cisplatin vs. ACY-1083:Cisplatin | Tukey's post hoc | < 0.0001 | -26.62, -10.05 |
| Fig. 5S | ACY-1083:PBS vs. ACY-1083:Cisplatin | Two-way ANOVA | Interaction: p = 0.0269; vehicle vs. ACY-1083: p = 0.1146; PBS vs. Cisplatin: p = 0.0270 |  |
| Fig. 5S | Vehicle:PBS vs. Vehicle:Cisplatin | Tukey's post hoc | 0.0138 | 1.591, 16.9 |
| Fig. 5S | Vehicle:Cisplatin vs. ACY-1083:PBS | Tukey's post hoc | 0.0435 | -15.49, -0.1805 |
| Fig. 5S | Vehicle:Cisplatin vs. ACY-1083:Cisplatin | Tukey's post hoc | 0.0434 | -15.49, -0.182 |
| Fig. 5T | ACY-1083:PBS vs. ACY-1083:Cisplatin | Two-way ANOVA | Interaction: p = 0.0138; vehicle vs. ACY-1083: p = 0.1157; PBS vs. Cisplatin: p = 0.0193 |  |
| Fig. 5T | Vehicle:PBS vs. Vehicle:Cisplatin | Tukey's post hoc | 0.0065 | 2.423, 17.4 |
| Fig. 5T | Vehicle:Cisplatin vs. ACY-1083:PBS | Tukey's post hoc | 0.0346 | -15.43, -0.4594 |
| Fig. 5T | Vehicle:Cisplatin vs. ACY-1083:Cisplatin | Tukey's post hoc | 0.0275 | -15.71, -0.7417 |
| Fig. 6I | ACY-1083:PBS vs. ACY-1083:Cisplatin | Two-way ANOVA | Interaction: p = 0.0004; vehicle vs. ACY-1083: p = 0.0012; PBS vs. Cisplatin: p = 0.0022 |  |
| Fig. 6I | Vehicle:PBS vs. Vehicle:Cisplatin | Tukey's post hoc | 0.0003 | -43.66, -15.12 |
| Fig. 6I | Vehicle:Cisplatin vs. ACY-1083:PBS | Tukey's post hoc | 0.0005 | 13.19, 41.73 |
| Fig. 6I | Vehicle:Cisplatin vs. ACY-1083:Cisplatin | Tukey's post hoc | 0.0002 | 16.29, 44.84 |
| Fig. 6J | ACY-1083:PBS vs. ACY-1083:Cisplatin | Two-way ANOVA | Interaction: p = 0.0020; vehicle vs. ACY-1083: p = 0.0065; PBS vs. Cisplatin: p = 0.0003 |  |
| Fig. 6J | Vehicle:PBS vs. Vehicle:Cisplatin | Tukey's post hoc | 0.0002 | -41.89, -15.26 |
| Fig. 6J | Vehicle:Cisplatin vs. ACY-1083:PBS | Tukey's post hoc | 0.0004 | 13.26, 39.89 |
| Fig. 6J | Vehicle:Cisplatin vs. ACY-1083:Cisplatin | Tukey's post hoc | 0.0013 | 9.553, 36.18 |
